# Integrating mental health into primary health care in Zambia: a care provider's perspective

**DOI:** 10.1186/1752-4458-4-21

**Published:** 2010-07-25

**Authors:** Lonia Mwape, Alice Sikwese, Augustus Kapungwe, Jason Mwanza, Alan Flisher, Crick Lund, Sara Cooper

**Affiliations:** 1 Department of Mental Health Nursing, Chainama College of Health Sciences, Lusaka, Zambia; 2 Department of Social Development Studies, Demography Division, University of Zambia, Lusaka, Zambia; 3 Department of Psychiatry and Mental Health, University of Cape Town, South Africa; 4 Research Officer, Mental Health and Poverty Project, Department of Psychiatry, Chainama College of Health Sciences, University of Zambia, Lusaka, Zambia

## Abstract

**Background:**

Despite the 1991 reforms of the health system in Zambia, mental health is still given low priority. This is evident from the fragmented manner in which mental health services are provided in the country and the limited budget allocations, with mental health services receiving 0.4% of the total health budget. Most of the mental health services provided are curative in nature and based in tertiary health institutions. At primary health care level, there is either absence of, or fragmented health services.

**Aims:**

The aim of this paper was to explore health providers' views about mental health integration into primary health care.

**Methods:**

A mixed methods, structured survey was conducted of 111 health service providers in primary health care centres, drawn from one urban setting (Lusaka) and one rural setting (Mumbwa).

**Results:**

There is strong support for integrating mental health into primary health care from care providers, as a way of facilitating early detection and intervention for mental health problems. Participants believed that this would contribute to the reduction of stigma and the promotion of human rights for people with mental health problems. However, health providers felt they require basic training in order to enhance their knowledge and skills in providing health care to people with mental health problems.

**Recommendations:**

It is recommended that health care providers should be provided with basic training in mental health in order to enhance their knowledge and skills to enable them provide mental health care to patients seeking help at primary health care level.

**Conclusion:**

Integrating mental health services into primary health care is critical to improving and promoting the mental health of the population in Zambia.

## Background

Over the last decade, Zambia has embarked on a radical transformation process aimed at creating a well functioning, cost effective and equitable district-based health care system [1]. Such reforms were in response to the government's growing awareness of the innumerable health challenges afflicting the nation. These reforms were based on a primary health care concept, as it was believed that most diseases in Zambia were either preventable or could be managed at the primary health care level [2]. Thus, primary health care was chosen as a vehicle through which to deliver health services to the population. A number of progressive health laws and regulations were devised, together with the Basic Health Care Package (BHCP). This Package is a set of carefully selected high impact interventions offered through the public health system at no out of pocket cost to the user or on a cost-sharing basis at different levels of the health care delivery system [2,3]. Selection of the interventions included in the BHCP was done through epidemiological analysis of those diseases that caused high morbidity and mortality rates in the country. A total of ten priority areas qualified for inclusion on the BHCP [4].

Mental health was not amongst the ten priority areas for health services in the BHCP. In fact, none of Zambia's strategic health plans and policy documents have addressed and incorporated mental health [4]. Although the burden of mental disorders is unknown in Zambia, hospital-based figures in Zambia suggest a prevalence rate of 3.61 and 1.8 per 10 000 population served by the hospitals catchment area for acute psychotic states and schizophrenia respectively. These are based on the number of existing cases for a particicular period of a year, per 10 000 of the population [5]. Furthermore, treatment for 200 000 people with mental disorders (of an adult population of 2 million) in Zambia is either lacking or provided in a fragmented and not evidence-based manner [6]. It is thus a cause for concern that mental health has been largely overlooked in Zambia, and not included within the Basic Health Care Package [5].

Mental health services appear to have been inadequately incorporated into the primary health care in Zambia, a problem shared with many other low-income African countries [7-9]. Although there are psychiatric units within seven general hospitals across the country, and the mental health policy in the country has made a commitment to integration, mental health services in Zambia are largely delivered at Chainama Hospital in Lusaka, the only mental health hospital in the country [10]. According to MHaPP Country Report [11], about 2667 patients per 100,000 population are admitted to Chainama and psychiatric units around the country. The total number of beds in Chainama is 210 excluding 167 (floor beds) which are not officially recognised by the Ministry of Health. Primary health care units (health centres) do not have any mental health plans, and are severely fragmented and unco-ordinated [12,13,10]. In these facilities, there are also inadequate psychotropic drugs, and the few staff that are available have either inadequate knowledge about mental health or they are unable to cope with the inclusion of people with mental health problems in their work schedule [10]. It has been observed that mental health referral services at the primary health care level have practically collapsed [5].

There has been widespread recognition of the benefits of and need for low and middle income countries to better integrate mental health within primary health care [7,14]. Integrating mental health into primary health care has been shown to reduce mental health care costs [15] and provide the best practice in the provision of treatment, rehabilitation and general care of psychiatric patients [16-18].

There is a dearth of research on mental health generally in Zambia [12,13,5] with currently no research having been conducted on issues around integration of mental health with primary health care. This current paper presents part of the data that was collected for a Knowledge Attitudes and Practices (KAP) survey that assessed the knowledge, attitudes and practices regarding mental health of general and mental health care providers' in Zambia. This survey formed part of the Mental Health and Poverty Project (MHaPP). The MHaPP, which is being conducted in four African countries: Ghana, South Africa, Uganda and Zambia, aims to investigate the policy level interventions required to break the vicious cycle of human poverty and mental ill-health, in order to generate lessons for a range of low-and middle-income countries [19]. The aim of this particular paper was to explore health care providers' views about mental health integration into primary health care. It sought to document whether there was support and/or resistance to such integration, possible reasons for such attitudes, and health care providers' recommendations regarding integration. This study will offer insights into how mental health could be better integrated into primary health care in Zambia and low-income African countries.

## Methods

A survey was devised and conducted in order assess the knowledge, attitudes and practices (KAP) regarding mental health of general and mental health care providers' in Zambia. The main objective behind this survey was to guide and inform the training that would be carried out amongst general and mental health care practitioners to better identify and manage common mental illness.

Data collection took the form of a questionnaire with both open and closed ended questions. The questionnaires were administered to selected health care providers who worked in Out-Patients departments. Seventy six questions were asked covering three topics namely: Knowledge, Attitudes and Practices. Knowledge and Practice included the following sub-headings; knowledge about causes of mental illness, knowledge about mental disorders and ability to treat, prescribe, and administer drugs while Attitudes involved; stereotypes, separatist and discriminatory attitudes, restrictiveness. The questions on which this paper is based formed part of the 'attitudes' component of the survey instrument which included questions on attitudes towards training, and attitudes towards integration. The guiding questions are shown in table 1

**Table 1:** Guiding questions on attitudes towards training and integration

	First Question	Follow-on Question
1	The Ministry of Health is soon to on training PHCstaff in identifying and management of mental disorders. How important do you think this is?	What are are the reasons for your answer?
2	The reason behind training of PHC in mental health is to integrate mental health into PHC system. How important do you think this is?	What are the reasons for your answer?
3	In your view, what type of impact will integration of mental health into PHC have on the provision of primary health care in general?	Please give the main reason for your answer.
4	What would be your opinion to the proposition that there are other more important areas of PHC other than mental health in which PHC staff should be trained.	Which are these areas

The questionnaire was piloted on fifteen health workers in Kafue District that was not part of the survey sites. Based on the findings from this pilot, the questionnaire was adapted and revised.

The data were collected from two purposively selected sites: Lusaka, representing an urban setting, and Mumbwa, representing a rural setting. These sites were selected as Ministry of Health pilot districts for integration of mental health into primary health care as well as for the purpose of representation of rural and urban scenarios. A total of 111 participants drawn from health facilities in the mentioned areas took part in the survey. Purposive sampling technique was used to select participants. Health workers from Out-Patients departments were selected because they see all incoming patients and refer them to respective departments depending on the patient's condition.

Participants were recruited from more than half the number of health centres in Lusaka. In Mumbwa they were recruited from health centres that were accessible. However, the health centres and clinics that participated were typical of all clinics in the sense that they are government financed and supervised health centres, and being served through the same basic health care package. The clinics also recruit categories of staff with similar levels of qualifications and training. In addition, almost all the health centres in Lusaka are placed in low density areas catering for similar characteristics of the population. The same applies to the rural health centres. Therefore the sample was representative of the districts from which participants were drawn. The data were collected between March and April 2009.

The quantitative data were entered into the excel data entry programme where numeric data were aggregated. Descriptive statistical analyses of relevant items were conducted.

The qualitative data were analysed using thematic analysis informed by Braun and Clarke [20] in order to identify common responses to the questions. The data were transcribed as word for word statements from participants. Initially, all responses were read several times over to gain a sense of the meaning as a whole. Long table analysis which is a low cost technology option was used to organise the data. Responses to questions from various groups of participants were coded in different colours, and were pasted onto flip charts. Each question was on a different flip chart, followed by responses from the different groups of participants identifiable by the colour codes. Independently identified codes were then compared, and where they addressed similar content, were combined into single categories. We then inductively looked for connections and relationships among the discrete codes, in order to uncover common themes and meanings across the entire data set. Themes were grouped according to the following: similarities; treatment; stigma and discrimination; human rights; training and resistance.

Permission to conduct this study was obtained from the Ministry of Health Directory of public health and research, and the District Directors of Health for the respective districts. Detailed information was provided to participants concerning participation and the consequence of the study. Participation was voluntary, and informed consent was obtained. For the purpose of anonymity, participants' names were omitted from the questionnaire.

## Results

### Social demographic characteristics

One hundred and one participants were took part in the survey. The age ranged between 19 and 65 years with the majority (41.4%) of them aged between 35 and 45 years. All participants were health care providers working in out-patients departments. Their work experience ranged from the newly graduated to those almost reached their retirement.

Table 2 shows age distribution, job title, work experience and qualification of health care staff surveyed. Most (79.2%) of the respondents were aged between 25 and 45 years. The job title (and percentage) of the respondents interviewed were as follows: Zambia Enrolled Nurses (29.7%), Zambia Registered Nurses (23.4%), Zambia Enrolled Psychiatric Nurse (6.3%), Zambia Registered Mental Nurses (1.8%), Clinical Officers Psychiatry (6.3%), Clinical Officers general (28.8%). Clinical Officers psychiatry and Registered Metal Health Nurses are front line staff in the delivery of mental health care in primary health care units in both long stay facilities and daily outpatient facilities. Clinical Officers prescribe psychotropic drugs which are then administered by nurses. Such staff members are lower than doctors, with the law inhibiting them from prescribing psychotropic drugs. In terms of work experience, more than two-thirds (81.1%) of those interviewed had been working as health care providers for more than four years with the majority (66.7%) having worked for more than five years. More than half (54.1%) of the respondents were certificate holders while the rest (45.9%) were diploma holders.

**Table 2:** Number and distribution of respondents by age group, job title, work experience and qualification

Characteristic	Frequency	Percent
**AGE GROUP**		
19-24	4	3.6
25-29	21	18.9
30-34	21	18.9
35-39	23	20.7
40-45	23	20.7
46-50	17	15.3
56-60	1	0.9
61-65	1	0.9
**TOTAL**	**111**	**100.0**
		
**JOB TITLE**		
Clinical Officer general	32	28.8
Clinical Officer Psychiatry	7	6.3
Zambia Registered Nurse	26	23.4
Zambia Enrolled Nurse	33	29.7
Zambia Enrolled Psychiatric Nurse	7	6.3
Zambia Registered Mental Nurse	2	1.8
Environmental Health Technologist	4	3.6
**Total**	**111**	**100.0**
		
**WORK EXPERIENCE**		
< 1 years	8	7.2
1-3 years	13	11.7
4 years	13	11.7
Five years	3	2.7
>5 years	74	66.7
**Total**	111	**100.0**
		
**QUALIFICATION**		
Certificate holder	60	54.1
Diploma holder	51	45.9
**Total**	**111**	**100.0**

### Attitudes towards integration

The results revealed a high degree of favorable attitudes towards the proposed integration of mental health into primary health care. As indicated in table 3, more than 71 percent (71.2%) of interviewees indicated that such integration was extremely important while 27 percent indicated that it was important. Overall, 98.2 percent were of the view that such integration was either extremely important (71.2%) or just important (27.0%) and this ranged from a high of 88.5 percent among Zambian Registered Nurses to 25.1 percent among Environmental Health Technologist who indicated that it was extremely important. The low percentage among Environmental Health Technologists could be due to the fact that only 4 respondents in this category of health care providers were captured in the study.

**Table 3:** Frequency and distribution of respondents by perceived degree of importance of integrating mental health into the primary health care system and perceived degree of importance of training in identification and management of mental disorders (broken down by job title)

*Job Title*	*Importance of Integration* N (%)*				*Importance of training* N (%)*			
	Extremely important	Important	Unimportant	Extremely Unimportant	Extremely important	Important	Unimportant	Extremely Unimportant
**Clinical Officer general (n = 32)**	20 (62.5)	10 (31.2)	0 (0.0)	2 (6.2)	18 (56.2)	9 (28.1)	0 (0.0)	5 (15.6)
**Clinical Officer Psychiatry (n = 7)**	4 (57.1)	3 (42.9)	0 (0.0)	0 (0.0)	5 (71.4)	2 (28.6)	0 (0.0)	0 (0.0)
**Registered Nurse (n = 26)**	23 (88.5)	3 (11.5)	0 (0.0)	0 (0.0)	15 (57.7)	9 (34.6)	0 (0.0)	2 (7.7)
**Enrolled Nurse (n = 33)**	23 (69.7)	10 (30.3)	0 (0.0)	0 (0.0)	25 (75.8)	6 (18.2)	2 (6.1)	0 (0.0)
**Enrolled Psychiatric Nurse (n = 7)**	6 (85.7)	1 (14.1)	0 (0.0)	0 (0.0)	5 (71.4)	1 (14.3)	0 (0.0)	1 (14.3)
**Registered Mental Health (n = 2)**	2 (100)	0 (0.0)	0 (0.0)	0 (0.0)	2 (100.0)	0 (0.0)	0 (0.0)	0 (0.0)
**Environmental Health Technologist (n = 4)**	1 (25.1)	3 (75.0)	0 (0.0)	0 (0.0)	2 (50.0)	2 (50.0)	0 (0.0)	0 (0.0)
**Total**	79 (71.2)	30 (27.0)	0 (0.0)	2 (1.8)	72 (64.9)	29 (26.1)	2 (1.8)	8 (7.2)

*NOTE: % of individuals within particular job description

### Rationale behind support for integration

The reasons different respondents gave for why mental health should be integrated into primary health care can be categorized into three main themes: Better detection and management of mental health problems; stigma reduction; and human rights.

#### 1. Better detection and managebment of mental health problems

A key theme advanced by most of the participants that supported integration was that integration of mental health services into primary health care will facilitate early detection of mental health problems and prevent complications. Many respondents indicated that integration would aid *"*e*arly intervention", "early detection" and that "Early diagnosis of mental illness patients will be facilitated" (Clinical Officer, General, Lusaka)*.

It was indicated further by many health care providers that many people avoid or delay seeking care and treatment at Chainama, the main mental hospital, as the institution is a major source of stigma. It was indicated that people with mental disorders would be more willing to access care and treatment at primary health care clinics, which do not have the same negative connotations attached:

*'Mental illness will not be let to reach advanced stage since early detection and treatment will be effected by both health facility and community, since some family members fear/avoid being associated with Chainama Hospital and end up delaying seeking attention' (Registered General Nurse, Mumbwa)*.

Similarly, another health care provider indicated:

*'People usually have a negative perception about Chainama and mostly people don't even go there if they are referred from health centres so by bringing this service nearer to their community and also being treated in the same facility just like any other patient, then people can be willing to get treatment from the centres without being stigmatized and discriminated against. Other problem can be identified apart from mental illness'(Registered General Nurse, Lusaka)*.

As alluded to in this last remark, integration of mental health with primary health care would also improve the general health care of persons with mental disorders.

Many participants also indicated that integration would facilitate the detection and management of mental disorders as mental health care would be brought closer to the communities. Numerous health care providers highlighted that many people have to travel long distances, and incur great costs to seek care at Chainama mental hospital. This was particularly emphasized by health care providers in rural areas. It was argued that mental health care would be more accessible and available if it was integrated into primary health care:

*'It will enable the mentally ill to receive care within easy reach in our community and lessen the cost of transport to other institutions like Chainama'(Enrolled Nurse, Mumbwa)*.

In addition, it was indicated that mental health workers are in short supply in comparison with other types of health workers. It was thus suggested that integration would mean that there would be many more staff available for the management and treatment of mental disorders, as general health care workers would be able to attend to patients. This was most aptly revealed by the following two statements:

*'All health care providers would receive training and will be ready to handle mental illness cases in the community and at health centre level, so there would be many more staff available'(Clinical Officer, Psychiatry, Mumbwa) *and

*'Patients will be receiving professional case management from all health workers'(Environmental Health Technologist, Mumbwa)*.

The rationale behind this was summarised by one participant as follows:

*'Primary health care system is the only health framework can bring health as close as possible to the communities'(Registered Mental Health Nurse, Lusaka)*.

There was also widespread agreement that integration would aid better detection and management of mental health problems because less people would go to Chainama mental hospital, and Chainama would be less congested and overcrowded. This would ensure that those patients who are admitted to the main mental hospital, would receive better and more comprehensive care. Many respondents indicated that integration will create "*less congestion at Chainama Hospital" as "'People with simple mental illness could be handled within the clinic instead of sending them to Chainama'(Clinical Officer, General, Lusaka)*.

##### 2. Stigma reduction

The issue of stigma and discrimination appeared to be prominent in many participants' responses around integration of mental health into primary health care. Numerous respondents who supported integration stated that stigma would be reduced if people with mental health problems were treated at primary health care units. It was not uncommon to hear statements such as "*once integrated they come out of stigma"(Enrolled Nurse, Lusaka) *and integration would *"lessen stigma"(Clinical Officer, General, Lusaka)) *and *"reduce negative attitudes"(Enrolled Nurse, Mumbwa)*. It was suggested that stigma would be reduced because people would start to *"regard mental illness as a disease like any other because they would be treated in the same facility just like any other patient"(Registered General Nurse, Mumbwa)*. Furthermore, one respondent indicated:

*'The community attitude will gradually change as they see mental patients are cared for and recover, rather than being locked away'*.

Many respondents shared this view that stigma would be reduced as many people would be able to receive care within their communities, rather than being institutionalized far away in Chainama.

#### 3. Human rights

Numerous participants stated that the government has made a clear commitment to providing health care to the population at the primary health care level. Some respondents said that over and above the benefits of integration, people with mental health problems possess an intrinsic right to treatment at this level of care, just like any other person seeking help for a health problem. They thought that integration of mental health at primary health care level would serve to uphold the human rights that people with mental health problems are thus equally entitled to. As one respondent indicated when stating that he supported integration:

*'Because mentally ill patients like any other patients deserve treatment and care from the primary health care system, in health centres'(Clinical Officer,General, Mumbwa)*.

This view was shared by another health care worker who stipulated:

*'The patients are all important and need equal rights and attention as early as primary care level'(Enrolled Nurse, Mumbwa)*.

Indeed, many respondents spoke about how people with mental disorders have the *"right to receive care at the primary level"(Enrolled Psychiatric Nurse, Lusaka)*, and the *"right to care without segregation"(Environmental Technologist, Mumbwa)*.

### Possible rationale behind resistance to integration

Although respondents who were against integration were not asked directly why they held such attitudes, some of the reasons could be hypothesized based on respondents responses to other questions such as the one asking about the importance participants attached to integrating mental health into primary health care. As such, it seems that fear and negative attitudes towards people with mental disorders may underlie some health care providers reservations regarding integration. Table 4 shows the degree to which respondents are comfortable to attend to or deal with patients with mental illness. The majority (68.4%) of health care providers interviewed admitted that they were extremely uncomfortable (19.8%) or uncomfortable (48.6%) attending to mentally ill people. Similarly, more than two thirds (62.1%) of the respondents indicated that they were either extremely uncomfortable (25.2%) or just uncomfortable (36.9%) dealing with mentally ill persons in general. Furthermore, as indicated in table 4, nearly half (44.1%) of respondents indicated that they find it hard to talk to someone with mental health problems, while 42.3% agreed that detention in a solitary place should be considered for people with mental illness. Also, more than two-thirds (67.5%) of the respondents strongly agreed (27.9%) or agreed (39.6%) with the notion that people with mental health problems should not be treated in the same health centre as general patients.

**Table 4:** Number and distribution of respondents by the degree to which they are comfortable to attend to or deal with mentally sick people

Degree of comfort	Attending to patients with mental illness		Dealing with mentally sick people	
	Frequency	Percent	Frequency	Percent
Extremely uncomfortable	22	19.8	28	25.2
Uncomfortable	54	48.6	41	36.9
Comfortable	31	27.9	37	33.3
Extremely comfortable	4	3.6	5	4.5
TOTAL	111	100.0	111	100.0

It thus seems that such attitudes may be informing certain reservations around potential integration between mental health and primary health care.

### Need for training

There was widespread agreement amongst respondents that better integration of mental health into primary health care required increased training in the identification and management of mental disorders. Responses are shown in Table 5 by category of health care provider. Most respondents (91%) indicated that training was either extremely important (64.9%) or important (26.1%). Only about 10 percent thought that such training was extremely unimportant (7.2%) or unimportant (1.8%). The positive attitude towards training cuts across all categories of health care providers captured in the study.

**Table 5:** Number and distribution of health workers by the degree to which they agree or disagree with statements regarding mentally sick persons

	*Strongly disagree*	*Disagree*	*Agree*	*Strongly agree*	*Undecided*	TOTAL
Find it hard to talk to someone with mental health problems	9(8.1)	37(33.3)	49(44.1)	13(11.7)	3(2.7)	111(100.0)
Detention in a solitary place should be considered for people with mental illness	20(18.0)	26(23.4)	47(42.3)	7(6.3)	11(9.9)	111(100.0)
Mental patients should not be treated in the same health center with other people	5(4.5)	24(21.6)	44(39.6)	31(27.9)	7(6.3)	111(100.0)
Mentally sick persons are entitled to the same attention in the health center as general patients	19(17.1)	16(14.4)	30(27.0)	45(40.5)	1(0.0)	111(100.0)
Chainama is the only place for people with mental illness	16(14.4)	29(26.1)	35(31.5)	28(25.2)	3(2.7)	111(100.0)

It was indicated that a lot of mental health problems currently go undiagnosed at the primary health care level because of inadequate knowledge and skills to identify and treat mental health problems. As one respondent indicated:

*'Most cases go unidentified and undiagnosed, so training in mental health at the primary level is long overdue'(Clinical Officer, Psychiatry, Mumbwa)*.

It was indicated that training would equip general health care workers with necessary skills to manage cases of mental illness appropriately. The rural population indicated that training in mental health would also prevent them from referring uncomplicated cases to Chainama hospital. As the following remark indicates:

*'Training will equip us to manage mental illness cases at clinic level and give adequate support'(Registered General Nurse, Lusaka)*.

## Discussion

This study explored integration of mental health into primary health care from the perspective of health care providers in two districts in Zambia. It documents their attitudes towards integrating mental health into primary health care, as well as some of the possible reasons for such attitudes. It also highlights health care providers' recommendations regarding integration. One of the key barriers around integrating mental health into primary health care that has been identified is that the views and concerns of health care providers around integration have not been fully taken into account [14]. This study thus seeks to speak to this gap. To the authors knowledge, it is also the only known study on mental health integration into primary health care in Zambia.

The results revealed a general willingness amongst health workers to have mental health added to their list of care responsibilities, and integrated with primary health care. A multitude of reasons were highlighted as to why integration would be beneficial. These included improving the detection and management of mental health problems as people would be more willing to access care, care would be brought closer to the communities, there would be more human resources and Chainama would be less congested. Furthermore, it was indicated that integration would help reduce the stigma surrounding mental illness, as people would receive care in the same facilities as other patients, rather than being institutionalized. Finally, some respondents indicated that integration was a human right for people with mental disorders.

Integrating mental health into primary health care has been shown to improve the diagnosis of mental disorders and uptake of treatment, as health care at the primary level is generally more accessible, available and less stigmatizing [14]. Furthermore, it has been shown that because up to 40% of patients attending primary care services will be suffering from the common mental disorders, the opportunities for prevention, both primary and secondary, are greater with integration [21].

However, while most respondents favoured integration, most also said that people with mental health problems should not be treated in the same health centre as general patients. Although it was not explored fully, it seems that resistance to integration may stem from the fear and negative attitudes some health care providers may have towards mentally ill persons. Indeed, fear and stigma, which are common amongst general health care providers [22-25], have been identified as some of the main obstacles preventing adequate mental health and primary health care integration[26]. In this regard, further work may be needed, not only in the provision of clinical skills, but also in providing education, changing attitudes and beliefs.

The World Health Organisation has been encouraging nations to have a deliberate policy to integrate mental health into primary health care since the Alma Ata International Conference on Primary Health Care in 1978. Despite the numerous health sector reforms that have taken place in Zambia that have been situated within a primary health care philosophy, mental health has been largely overlooked in these reforms. Indeed, the Zambian health service's priority is placed on communicable diseases, at the expense of non-communicable diseases. Jenkins and Stratdee [14] emphasize however that ignoring the burden of disease arising from mental health problems can be expensive in terms of absenteeism, increased demand for health services, and low productivity, among others.

It is with the above background in mind that the present study suggests restructuring of mental health services through its integration into primary health care and involvement of the community in the management of mental health problems and promotion of mental health [27]. Researchers in other settings argue for what is termed 'comprehensive integrated primary mental health care', a concept that takes into account the complexity of the interaction of biological, cultural, social and psychological factors in mental health [27,28]. Comprehensive changes such as reforming the mental health system require greater strength than would be needed to change a segment of it [27].

Bearing in mind the commitment by government contained in the Zambian mental health policy [10] that mental health should be integrated at primary health care level, it would be expected that the first step towards integration should be the inclusion of mental health on the BHCP. Like any other intervention accessible at the primary health care level, mental heath can also be easily accessed. The present study findings revealed the need for mental health problems to be detected earlier. Therefore the importance for the reform programme to ensure mental health services are made practically available within primary health cannot be overemphasised. This may be appropriate because it is easier for many people to access health care from this level given that health facilities are within their reach, and therefore affordable in terms of transport costs. The issue of prohibitive transport cost to take a patient to Chinama has been one of the common concerns for most of the participants of the present study.

The recommendations of this study are consistent with Orley and Sartorius' [29] suggestion that health care providers should be able to attend to every patient's psychological dimension of health beside the physical ailment that has led the patient to seek health help. If they do not possess the skill to attend to people with mental health problems, health providers should at least have the skill to identify the problem and refer to their colleagues who are competent to provide mental health interventions. In this case, every health worker in out-patient clinics or even on the ward should be provided with basic training to enable them to explore the mental health status of a person who comes to the clinic for a physical assessment and be able to identify a mental health problem. A conducive environment with knowledgeable and skilled staff on hand to help people presenting with mental health problems may serve to encourage the people to seek help when they need it, thus reducing the prevalence of mental health problems.

In this way, the health system would be upholding the holistic concept and person centred approach to care that WHO has recommended in its mental health programmes. Given the current inadequacy in human resource in Zambia in general and at primary health care level in particular, it is appropriate to build capacity so that people with mental health problems can be seen and cared for by health care providers who are not specialised in mental health but have basic knowledge and skills.

## Conclusion

It is thus clear that in principle, primary health care workers in Zambia appear to support integration of mental health into primary health care, but many still have stigmatising attitudes towards the mentally ill. Consequently, further work is needed, not only in the provision of clinical skills, but also in providing education to change attitudes and beliefs. Integrating mental health into primary health care is however ultimately going to require increased consensus, commitment and political will within government to place mental health on the national agenda and secure funding for the sector. This is essential if the country is to realize the many of the ideals enshrined in the progressive health reforms undertaken over the last decade. As indicated by the World Health Organization, ultimately, there is "no health without mental health" [18].

## Competing interests

The authors declare that they have no competing interests.

## Authors' contributions

AF and CL conceived of the study, and participated in its design and coordination and helped to draft the manuscript. LW and SC drafted the manuscript. AS, AK and AS were involved in revising the manuscript critically for important intellectual content. All authors read and approved the final manuscript.

## References

[B1] Ministry of Health National health policies & strategies: Health reform 1992 Lusaka: Ministry of Health

[B2] Central Board of Health Action planning handbook 1st level referral hospital 2001 2 Lusaka: Central Board of Health

[B3] WHO: World Health Report Health systems: improving performance 2000 Geneva, Switzerland: World Health Organisation

[B4] Fifth National Development Plan Government of the Republic of Zambia: Ministry of Finance & National Planning Lusaka: Ministry of Finance and Economic Development

[B5] Mayeya J Chazulwa R Mayeya P Mbewe E Mwape-Magolo L Kasisi F Zambia mental health country profile International Review of Psychiatry 2004 16 1-2 63 72 10.1080/09540260310001635113 15276939

[B6] WHO World Mental Health Survey: World Mental Health Surveys Prevalence, severity, and unmet need for treatment of mental disorders in the World Health Organisation JAMA 2004 291 2581 2590 10.1001/jama.291.21.2581 15173149

[B7] Horton R Launching a new movement for mental health Lancet 2007 370 306 10.1016/S0140-6736(07)61243-4 17804065

[B8] Jacob KS Sharan P Mirza I Garrido-Cumbrera M Seedat S Mari JJ Mental health systems in countries: Where are we now? Lancet 2007 370 1061 1077 10.1016/S0140-6736(07)61241-0 17804052

[B9] Knapp M Funk M Curran C Prince M Grigg M McDaid D Economic barriers to better mental health practice and policy Health Policy Plan 2006 21 157 170 10.1093/heapol/czl003 16522714

[B10] Ministry of Health Mental healthy policy 2005 Lusaka: Ministry of Health

[B11] Mental Health and Poverty Project Mental Health Policy Development and implementation in Zambia: A situational analysis Zambia Chapter 2008

[B12] Gleisner J The state of Zambian psychiatric services: Where to from now? Lusaka 2001

[B13] Gleisner J What causes more destruction, AIDS or AID? Psychiatry in Zambia Australian Psychiatry 2002 10 suppl 2 166 167 10.1046/j.1440-1665.2002.00426.x

[B14] Jenkins R Strathdee G The Integration of Mental Health Care with Primary Care International Journal of Law and Psychiatry 2000 23 277 291 10.1016/S0160-2527(00)00037-6 10981272

[B15] Knapp M Chisholm D Astin J Lelliott P Audini B The cost consequences of changing the hospital-community balance: the mental health residential care study Psychological Medicine 1997 27 681 692 10.1017/S0033291796004667 9153688

[B16] Cohen A The effectiveness of mental health services in primary care: the view from the developing world 2001 Geneva: World Health Organisation

[B17] Mynors WL Problem solving treatment: evidence for effectiveness and feasibility in primary care International Journal of Psychiatric Medicine 1996 26 249 262 10.2190/0HVY-CD2F-0KC7-FVTB 8976466

[B18] Prince M Patel V Saxena S Maj M Maselko J Philips MR Rahman A Global Mental Health: No health without mental health Lancet Series 2007 1 859 877 10.1016/S0140-6736(07)61238-0 17804063

[B19] Flisher A J Lund C Funk M Banda M Bhana A Doku V Mental health policy development and implementation in four African countries Journal of Health Psychology 2007 12 505 516 10.1177/1359105307076237 17440000

[B20] Braun V Clarke V Using thematic analysis in psychology Qualitative Research in Psychology 2006 3 77 101 10.1191/1478088706qp063oa

[B21] Murray J Jenkins R Prevention of mental illness in primary care International Review of Psychiatry 1998 10 154 157 10.1080/09540269874664

[B22] Byrne P Stigma of mental illness and ways of diminishing it Advances in Psychiatric Treatment 2000 6 66 72 10.1192/apt.6.1.65

[B23] Sartorius N Iatrogenic stigma of mental illness British Medical Journal 2002 324 1470 1471 10.1136/bmj.324.7352.1470 12077020 PMC1123430

[B24] Schulze B Stigma and mental health professionals: A review of the evidence on an intricate relationship International Review of Psychiatry 2007 19 suppl 2 137 155 10.1080/09540260701278929 17464792

[B25] Thornicroft G Rose D Kassam A Discrimination in health care against people with mental illness International Review of Psychiatry 2007 19 suppl 2 113 122 10.1080/09540260701278937 17464789

[B26] Gask L Overt and covert barriers to the integration of primary and specialist mental health care Social Science and Medicine 2005 61 1785 1794 10.1016/j.socscimed.2005.03.038 15913862

[B27] Petersen I Comprehensive integrated primary mental health care for South Africa. Pipedream or Possibility Social Science and Medicine 2000 51 321 334 10.1016/S0277-9536(99)00456-6 10855920

[B28] Robertson B Zwi R Ensink K Malcolm C Milligan P Motinho D Uys L Vitus L Watson R Wilson D Foster D, Freeman M, Pillay Y Psychiatric service provision Mental health policy issues for South Africa 1997 Cape Town: Medical Association of South Africa Multimedia Publications 69 93

[B29] Orley J Sartorious N Shepherd M, Wilkinson G, Williams P Mental illness in primary health care in developing countries Mental illness in primary health care in developing settings 1986 London: Tavistock Publications 195 200

